# Organellar genome dynamics of exogenous stages of *Eimeria tenella*

**DOI:** 10.1186/s13071-024-06498-w

**Published:** 2024-10-13

**Authors:** Perryn S. Kruth, Taylor Lane, John R. Barta

**Affiliations:** University of Guelph, Guelph, ON Canada

**Keywords:** Apicomplexa, Coccidiosis, Oocysts, Apicoplast, Eimeria, DNA copy number variations

## Abstract

**Background:**

Coccidia are a group of intracellular protozoal parasites within the phylum Apicomplexa. *Eimeria tenella*, one of the species that cause intestinal coccidiosis in poultry, can cause significant mortality and morbidity. Diploid oocysts of *Eimeria* species are shed in the feces of an infected host and must sporulate to achieve infectivity. This process results in eight haploid infectious units, called sporozoites, held within a single oocyst. Each *Eimeria* spp. parasite possesses a single apicoplast and a single mitochondrion, both of which carry multiple copies of their respective organellar genomes. Reports of copy numbers of organellar genomes have varied widely.

**Methods:**

We report the application of quantitative polymerase chain reaction (qPCR), supported by next-generation sequencing, for the quantification of the extranuclear genomes relative to the nuclear genome over the course of sporulation and following its completion.

**Results:**

At 64 elapsed hours, 93.0% of oocysts were fully sporulated; no increase in percent sporulation was observed after this time. Apicoplast relative genome copy number showed several significant shifts up to 72 elapsed hours, after which no significant shifts were observed. Oocysts were shed with approximately 60% the amount of apicoplast DNA present at 72 h, after which point no significant shifts in apicoplast genome relative abundance occurred. Mitogenome relative copy number showed only two significant shifts, from 16 to 24 elapsed hours and from 24 to 32 elapsed hours. Oocysts were shed with approximately 28% the amount of mitochondrial DNA that was present at the time sporulation was deemed morphologically complete, at 64 elapsed hours.

**Conclusions:**

The characterization of the dynamics of genome abundance in exogenous stages sheds new light on the basic biology of *Eimeria* spp. and supports the use of extranuclear targets for molecular modes of parasite quantification and identification with improved sensitivity and accuracy.

**Graphical Abstract:**

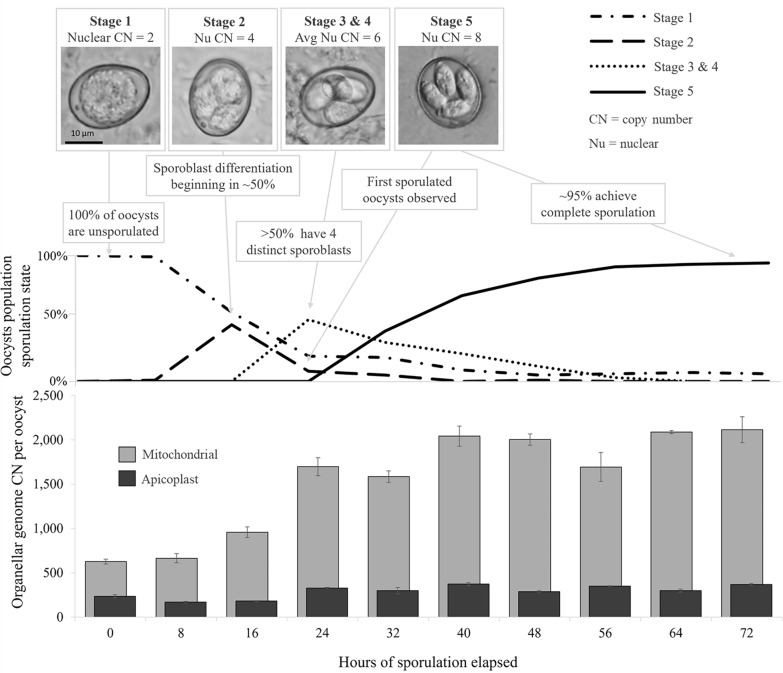

**Supplementary Information:**

The online version contains supplementary material available at 10.1186/s13071-024-06498-w.

## Background

Sporulation is the process by which freshly shed *Eimeria* spp. develop into the infectious form, comprising eight haploid infectious units (sporozoites), within a single oocyst [1–4]. Early in sporulation, meiosis produces four haploid progeny cells—sporoblasts—around which sporocyst walls form. A subsequent round of mitosis produces two haploid parasites inside each of the four sporocysts, for a total of eight infectious sporozoites per oocyst [1].

Most members of the phylum Apicomplexa possess two extranuclear (organellar) genomes, located in the mitochondrion and in the apicoplast [5–7]. The organellar genomes are present in multiple copies and are inherited via the macrogamete (maternally) [7, 8]. Gene content of the ~30–50 kb apicoplast genome is involved primarily in its own maintenance and expression and includes genes encoding proteins, transfer RNA (tRNA), and ribosomal RNA (rRNA) [9–11]. The mitochondrial genome ranges from 6 to 11 kb [12–15] and includes three protein encoding genes and a set of highly fragmented genes encoding rRNA, with tRNA encoding sequences completely absent [7, 13, 16, 17].

The unsporulated oocyst (zygote) contains a single diploid nuclear genome, with one haploid copy inherited from each of the macro- and microgametes [18]. This brief state of diploidy is characteristic of the lifecycles of members of the phylum [18]. The unsporulated oocyst also possess an undermined number of copies of each of the apicoplast and mitochondrial genomes. Upon completion of sporulation, the oocyst contains eight sporozoites that together contain eight haploid nuclear genomes and eight “organelles worth” of each of the apicoplast and mitochondrial genomes. Studies of *Toxoplasma gondii* have shown that replication of the apicoplast genome is completed prior to the end of G_1_, and studies using *Plasmodium falciparum* have shown mitogenome replication to begin at the same time as replication of the nuclear genome, however, to be completed more quickly [19]. Shifts in mitochondrial genome copy number (GCN) are associated with development and changing metabolic needs across eukaryotes [7, 19–23]. Chloroplasts, the evolutionary cousins to apicoplasts, possess genomes with copy numbers that also shift with development [24, 25]

*Eimeria tenella* is one of several *Eimeria* spp. that cause coccidiosis, a cosmopolitan disease with major impacts on the commercial poultry industry [1, 2, 26]. Under optimal conditions (sufficient oxygen, moisture, and temperature), sporulation of *E. tenella* has been reported to require approximately 48 h [1]. Once sporulation is complete, parasites enter a hypobiotic state and may remain infectious for months [3, 26, 27].

With the work described herein, we aim to determine the relative copy numbers (CNs) of the mitochondrial and apicoplast genomes of *E. tenella* throughout and following sporulation, in relationship to the nuclear genome. Two multicopy genome sequences were used to determine nuclear genome copy number: the random amplified polymorphic DNA-sequence characterized amplified region (RAPD-SCAR) Tn-E03-1161 and 18S ribosomal DNA. We predicted the organellar GCN per sporozoite to be different from that per macrogamete, and the ratio of organellar-to-nuclear genomes to remain constant in oocysts after sporulation was complete. Even when collected at a single time point, oocysts in stocks do not show completely synchronous sporulation [26]; we expected molecular data reflecting relative abundance of organellar genomes to be clouded to some degree by this lack of total synchronicity and by the expected small proportion of oocysts that do not successfully sporulate [28–31].

## Methods

### Production and recovery of oocysts

Freshly propagated *E. tenella* oocysts (Guelph strain) were used to infect eight chickens by oral gavage, with 20,000 oocysts administered to each bird. Eight days post infection, the birds were killed, and ceca were removed. Cecal contents, primarily consisting of oocyst cores, were collected and oocysts were cleaned and enumerated via standard protocols [32, 33]. Oocysts were incubated at 21 °C with agitation in potassium dichromate solution (K_2_Cr_2_O_7_; 2.0% w/v).

### Time point sampling

**Fig. 1:**
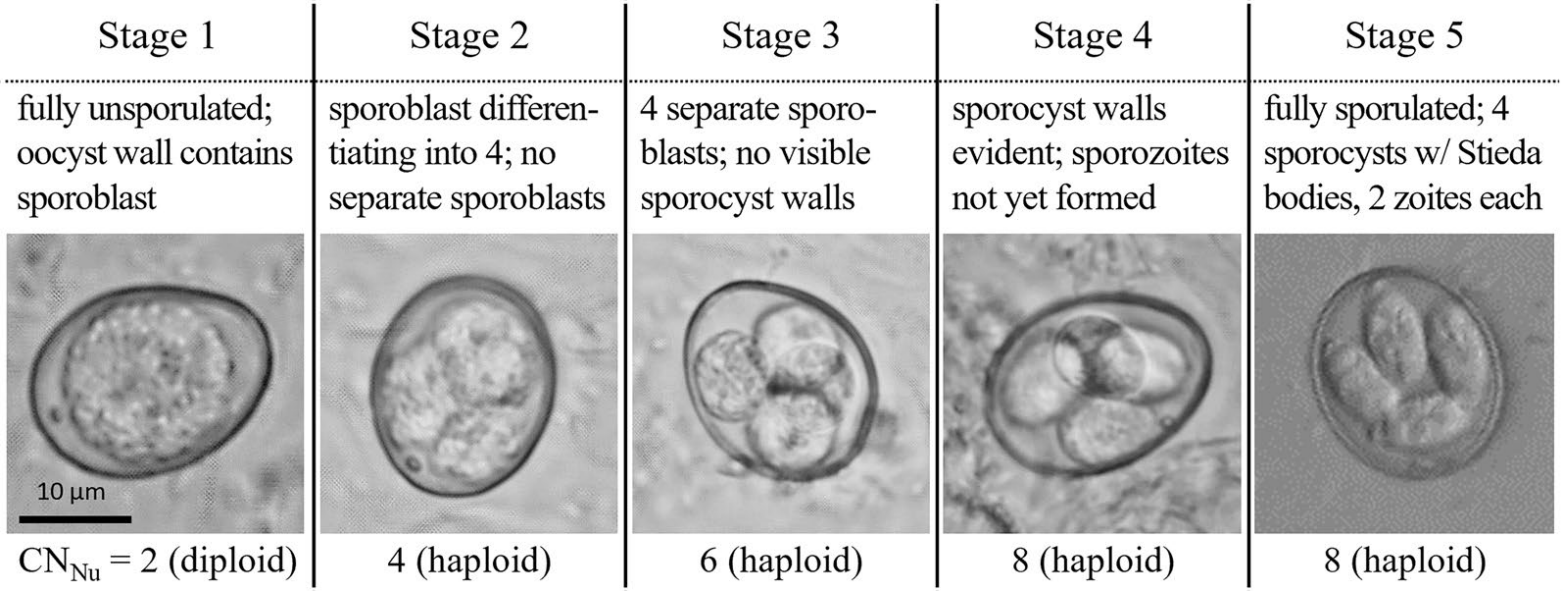
*Eimeria tenella* oocysts at sporulation stages 1–5, based on Al-Badri and Barta [29]. Estimated nuclear genome copy number (CN_Nu_) shown below each image. Stage 1 is characterized by the presence of a single, round sporoplast. In stage 2, the separation of the sporoblast into four can be visualized. In stage 3, there are four separate sporoplasts; the status of DNA replication cannot be visually determined, and CN_Nu_ of six is based on 50% oocysts with a CN_Nu_ of four and 50% with a CN_Nu_ of eight. Visualization of sporocyst walls but not sporozoites is possible during stage 4. Stage 5 oocysts are fully sporulated; they contain 2 sporozoites in each of 4 sporocysts that feature prominent Stieda bodies

Sampling occurred at 8 h intervals from immediately after the oocysts were cleaned (0 elapsed hours) to 72 elapsed hours and then at 88, 136, 235, 381, 523, 673, and 835 total elapsed hours. The complete sampling period spanned ~35 days. At each sampling time point, the percentage of oocysts identified at each sporulation stage was determined by microscopic examination of a subsample of oocysts at 100× magnification. Oocysts were assigned a sporulation stage from 1 to 5, based on descriptions by Al-Badri and Barta [27] (Fig. 1). Concurrent with sporulation state assessment, two aliquots of 7.0 × 10^5^ oocysts were pelleted via centrifugation, and the supernatant was removed prior to freezing at −80 °C for later isolation of DNA.

### DNA recovery

Samples were thawed for 10 min at room temperature. Oocysts were washed in 500 μL nuclease free water (NFW) and repelleted via centrifugation. Oocysts were resuspended in 100 μL DNAzol™ (Molecular Research Center, Cincinnati, Ohio) and ~0.4 g of 0.5 mm glass beads were added to each pellet. Oocysts were disrupted mechanically in three, 30 s bursts at 6000 rpm in a Precellys^®^ bead mill homogenizer (Bertin Technologies, Montigny-le-Bretonneux, France), with 5 s pauses between bursts. An additional 900 μL DNAzol™ was added to each sample before incubating for 30 min at 21 °C with light agitation. DNA extraction protocol continued as specified by the DNAzol™ manufacturers. Recovered DNA was resuspended in 50 μL NFW and was quantified using a Qubit^®^ Fluorometer and Qubit^®^ dsDNA BR Assay Kit (Thermo Fisher Scientific, Ottawa, ON, Canada). The DNA quality was assessed spectrophotometrically using a Nanodrop 2000™ instrument (NanoDrop, Wilmington, DE).

### qPCR target selection and primer design

A single quantitative polymerase chain reaction (qPCR) primer set was designed to target the apicoplast genome, and two primer sets were designed to target each of the nuclear and mitochondrial genomes. The primers were designed manually using Geneious Prime (2020.20.5), using publicly available *E. tenella* genomic sequences as reference (see following paragraph and Table 1) and for use with PowerUP™ SYBR™ Green Master Mix (Applied Biosystems, Waltham, MS), as per manufacturer’s recommendations. The DNA fold within Geneious Prime (2020.2.5) was used to ensure no major tendencies for secondary structure formation either within a single primer, self–self, between forward and reverse primers of one primer set or within the amplicon.

**Table 1:** Quantitative polymerase chain reaction primer sets targeting nuclear 18S ribosomal DNA (primer sets R2 and R3), nuclear Tn-E03-1161 sequence (S1 and S3), mitochondrial genome (M1 and M2), and apicoplast genome (P1) of *Eimeria tenella*

Primer name	Sequence (5′ to 3′)	Reference sequence	Melt temperature (T_m_)
R2_F	AACTCACCAGGTCCAGACATG	ETU67121.1	61.7 °C
R2_R	GATCACTCCACCAACTAAGAACG	ETU67121.1	61.0 °C
R3_F	CTCGGCTTATTTCCGGTAGC	ETU67121.1	60.6 °C
R3_R	TTATTCCATGCTGCAGTATTCAGG	ETU67121.1	61.0 °C
S1_F	GCGTTGTTGGCTATCGTAGG	AY571629.1	61.2 °C
S1_R	AGAGTGGACGGAAGATGAACG	AY571629.1	61.9 °C
S3_F	CTATGAAGCACCTGTGTACTGC	AY571629.1	61.0 °C
S3_R	CTGTAGTCGGCAATAGCGTTC	AY571629.1	61.1 °C
M1_F	GGGTTCGTGTTGTTGCATTAGAG	PP464288.1	62.2 °C
M1_R	CACCAAAAAGACCTGGCATAACTAC	PP464288.1	62.1 °C
M2_F	AAGTATCCCTGACTGGATTCTGC	PP464288.1	61.8 °C
M2_R	TGGACCAAGAGAAGCTGTGAG	PP464288.1	61.7 °C
P1_F^a^	CACCCGTACTAAAACTGACACAAG	AY217738	61.6 °C
P1_R	TTACAGGATTATTTTGCCGAGTTCC	AY217738	61.4 °C

^a^primer P1_F was modified from Biddeau et al. [36]

_F (suffix) is the forward qPCR primer

_R (suffix) is the reverse qPCR primer

The RAPD-SCAR sequence Tn-E03-1161 [34, 35] and ribosomal small subunit rDNA (18S rDNA) were chosen as nuclear targets. Primer sets S1 and S3 targeted Tn-E03-1161 (reference sequence, GenBank AY571629.1), and R2 and R3 targeted 18S rDNA (ETU67121.1). A single primer set (P1) was designed to target apicoplast 26S rDNA, which is present in two copies in the apicoplast genome (AY217738). The forward primer was modified from Biddau et al. [36] and was originally published as TogoCr29qPCR-F (5′-GCCCGTACTAAAACTGACACAAG-3′). Two primer sets were designed to target the mitochondrial genome (PP464288.1): M1 [targeting cytochrome *c* oxidase subunit I (*COI*)] and M2 (targeting an area that spans rDNA fragment LSU3 and part of rDNA fragment LSUB). Both target sequences exist once per mitochondrial genome. Table 1 includes qPCR primer sequences, melting temperatures, and accession numbers of reference sequences used.

Due to the lack of robust reference genome sequences for *E. tenella* at the time of experimentation, neither the sequence copy number (SCN) of 18S rDNA nor that of Tn-E03-1161 were known. Following experiment completion, a highly resolved reference genome for *E. tenella* was published [37]. The designed qPCR primer sets were tested against the 15 newly published chromosome sequences and two organellar genome sequences (HG994961.1 to HG994977.1) using “Test with saved primers” in Geneious Prime (2020.2.5; allow one mismatch and search for product sizes between 50 and 500 bp). The reference sequences used in designing the primer sets targeting 18S rDNA (ETU67121.1) and Tn-E03-1161 (AY571629.1) were additionally queried against the reference genome using BLAST.

### Primer validation and construction of a control plasmid

Primer technical validation, including the establishment of standard curves using a plasmid construct containing the qPCR target sequences is described in Additional file 1: >Supplementary Text 1 and Additional file 1: Supplementary Text 2. The primer set characteristics established via standard curves, including amplification efficiencies and *y* intercepts, are included in Additional file 1: Supplementary Table 1.

### Assessment of nuclear, apicoplast, and mitochondrial genome target abundance by qPCR

All primer sets were initially run at least once (in triplicate qPCR wells) with each time point sample. Subsequent repeat quantification of targets of interest in time point DNA samples used only M2, P1, R2, and S1 (all with triplicate qPCR wells). Only data generated from these four primer sets were used in determining the ratios of organellar to nuclear genomes reported for all time points.

Quantitative PCR components and cycling conditions were the same as used in establishment of standard curves, as follows: a total reaction volume of 10 μL per well contained 1× PowerUP SYBR Master Mix (Thermo Fisher Scientific, Waltham, MA) 600 nM forward primer and 600 nM reverse primer, and nuclease free water (NFW). Templates were diluted to 17–20 ng/μL, and 1.6 μL of template DNA was used per reaction. Each primer set was additionally run with a negative control (nuclease free water in place of template) and with three concentrations of a calibrator/positive cloned control sequence that contained one copy of each of the qPCR targets (resulting in 2.76 × 10^8^, 2.76 × 10^6^, or 2.76 × 10^4^ target copies per reaction). The reactions were conducted with a QuantStudio™ 7 Pro Real-Time PCR System (Applied Biosystems, Waltham, MA) in MicroAmp™ EnduraPlate™ Optical 384-Well Clear Reaction Plates (Applied Biosystems) with the following cycling conditions: UDG deactivation at 50 °C for 2 min, dual-lock DNA polymerase activation at 95 °C for 2 min; followed by 40 cycles of template denature at 95 °C for 15 s and a combined anneal/extend step at 60 °C for 1 min. The following melt program was used: 95 °C for 15 s (ramp rate: 1.6 °C/s), 60 °C for 1 min (ramp rate: 1.6 °C/s), 95 °C for 15 s (ramp rate: 0.1 °C/s). The cover temperature was set to 105 °C.

Design and Analysis Software Version 2.6.0 (Applied Biosystems) was used for preliminary analysis, including inspection of melt curves and triplicate well C_q_ variation, and to confirm absence of signal in no-template-control wells. All further analysis was executed in Excel [38]. Target SCNs were calculated for each well from C_q_ values, using primer regression parameters determined from standard curves (see Additional File 1: Supplementary Table 1 and Supplementary Formula 1).

### Whole-genome next-generation sequencing and read mapping

Twenty μL of DNA from eight of the time point samples (hours elapsed: 0, 8, 16, 24, 32, 64, 381, and 835 h), with concentrations ranging from 149–463 ng/μL and purity (A_260/280_) ranging from 1.90 to 2.01, was submitted to Lab Services of the Agriculture and Food Laboratory at the University of Guelph for library prep and next-generation sequencing (NGS). Sequencing libraries were generated with ~1 kb fragments. Two sequencing lanes were used, with four samples run together in equivalent ratios per lane. Paired-end 300 bp reads were generated using an Illumina MiSeq System (Illumina Inc., San Diego, CA).

Reads from each of the eight timepoints were paired, trimmed and filtered for Q30 and aligned to the *E. tenella* reference genome (HG994961.1–HG994977.1) using “map to reference” from within Geneious Prime (2023.1.2; advanced settings: maximum gaps per read of 10%, maximum gap size of 5, word length of 25, index word length of 15, maximum mismatches per read of 25%, and maximum ambiguity of 4). Depth of coverage (DOC) of each nuclear chromosome and of the mitochondrial and apicoplast genomes was calculated (“Statistics” section).

To further examine the ratio of 18S rDNA sequences to Tn-E03-1161 sequences, a reference sequence for NGS read mapping was generated by concatenating the 10 *E. tenella* reference genome 18S rDNA BLAST hits (query, GenBank: ETU67121), the six Tn-E03-1161 BLAST hits (query, AY571629), and the sequences of three *E. tenella* putatively single-copy reference genes: *HSP90*, *MIC1*, and β-tubulin [39–42], which are further described in Additional file 2: Supplementary Text 3. The 300 bp upstream and downstream of each of these elements was included in the concatenated sequence (sequence total length of 43,741 bp). All NGS reads that had previously mapped to *E. tenella* were pooled (for a total of 3,749,048 paired reads) and were mapped to the 43,741 bp reference sequence using Geneious Mapper from within Geneious Prime (2020.2.5; settings: map multiple best matches randomly, minimum overlap of 16, word length of 10, maximum mismatches per read of 2%, and maximum ambiguity of 4). The DOC of each element was determined (“Statistics” section).

### Statistics

#### Sporulation

The proportion of oocysts at each sporulation stage at each experimental time point was calculated from a sample of 100 oocysts, with 95% confidence intervals (CI) determined from standard error of samples proportions.

#### NGS depth of coverage from mapped reads for estimation of organellar genome abundances relative to the nuclear genome

Average DOC and standard deviation (SD) of DOC for each of the organellar genome NGS assemblies at each of the sequenced time points were determined via “Nucleotide Statistics” from within Geneious Prime (2020.2.5) and via Excel (see Additional file 2: Formula 2). The nuclear genome depth of coverage was calculated as the weighted average of individual chromosome depths of coverage. The SD of nuclear genome depth of coverage was calculated from the pooled SDs of each of the chromosome depths of coverage.

#### Nuclear target SCN estimation from NGS read mapping

To estimate the SCN of the nuclear target 18S rDNA, the average depth of coverage for 18S rDNA was calculated using the sum of all NGS reads that mapped to any of the 10 BLAST hits in the reference genome. Similarly, depth of coverage for Tn-E03-1161 was calculated from the sum of reads that mapped to any of the six BLAST hits in the reference genome. Ratios of the depth of coverage for 18S rDNA and for Tn-E03-1161 to the overall depth of coverage for the three single-copy genes were determined, with 95% confidence interval calculated from the extremes of the 95% confidence intervals of the Tn-E03-1161 and 18S rDNA CNs; see Additional file 2: Supplementary Table 2 for further details.

#### qPCR overall performance and calculation of absolute target CNs

Copy numbers of the S1, P1, M2, and R2 targets in control reactions and experimental sample reactions were calculated for each qPCR determined C_q_ value. To assess replicate well precision, the coefficient of variation of target CNs was calculated for technical triplicates of experimental samples and for standards. Average percent accuracy was calculated by dividing the SCN obtained from C_q_ interpretation by the known target SCN in control wells [(SCN determined from C_q_ value)/(known target SCN) × 100%].

#### Calculation of SCN of qPCR targets from C_q_-calculated absolute SCNs, relative to S1

At each time point, the ratio of the SCN for each technical repeat well for the P1, M2, and R2 targets to the average SCN of S1 was calculated. Ratio confidence intervals were calculated at 95% from the upper and lower extremes of the confidence intervals for the individual target copy numbers at each time point [95% CI for the copy number of M2, relative to S1 = (low end of 95% CI for M2 SCN)/(high end of 95% CI for S1)–(high end of 95% CI for M2 SCN)/(low end of 95% CI for S1)].

The calculation of CNs of the mitochondrial genome, apicoplast genome, and nuclear 18S rDNA, relative to the nuclear genome copy number from qPCR determined target relative abundances, and the S1 target was estimated to be present in the nuclear genome in 10 copies (“Results” section); the nuclear GCN was, therefore, calculated for each qPCR well as the calculated SCN of the S1 target, divided by 10. The average of technical replicate SCNs and the SD was determined for each time point. 18S rDNA SCN per genome was calculated by dividing the SCN of the R2 target by the average GCN calculated from S1 from the same experiment time point. Copy numbers of organellar genomes were determined as follows: the apicoplast GCN was calculated relative to a single nuclear genome by first subtracting the nuclear GCN from the P1 SCN at the matched time point (to adjust for the single P1 target that existed in the nuclear genome). Subsequently, the result was divided by two (to account for the P1 target being present in two copies within the apicoplast genome) and then divided by the average nuclear GCN from the same time point. The mitochondrial GCN was calculated relative to a single nuclear genome by dividing the M2 SCN by the average nuclear GCN for that time point. Finally, the mean ratio was calculated for each organellar to nuclear genome at each time point, with 95% confidence intervals calculated from the SD of the replicate calculated ratios.

To determine if shifts in mitochondrial genome relative abundance from one experimental time point to the next were statistically significant, a one-way repeated measures analysis of variance (ANOVA) was performed.

## Results

### Oocyst time point sampling

**Fig. 2:**
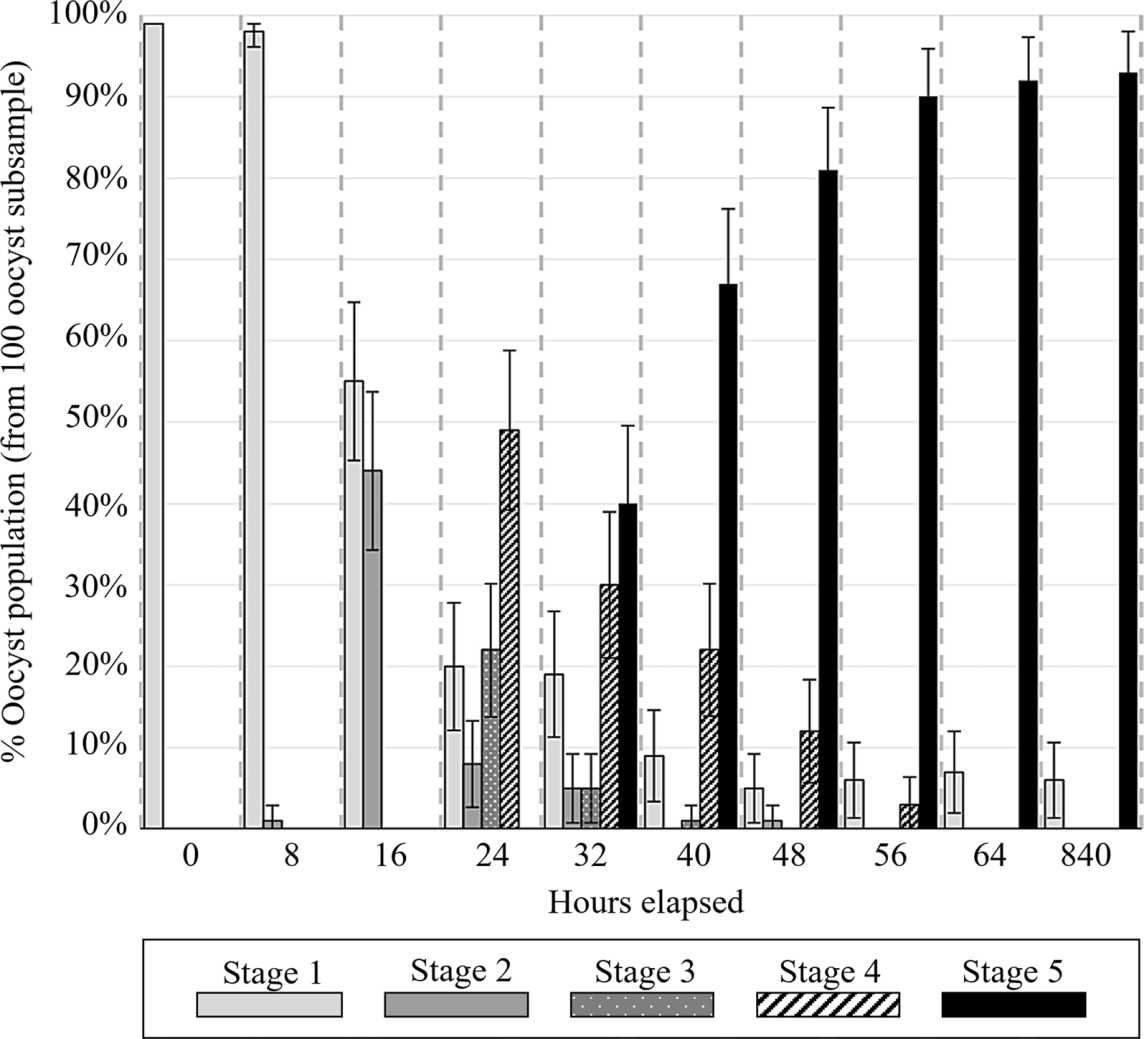
Percentage of *Eimeria tenella* oocysts at each sporulation stage, from zero elapsed hours (freshly collected from ceca) through to completion of sporulation (by 64 h) and at 835 h (~35 day) after collection. The error bars indicate 95% confidence intervals calculated from standard error of sample proportions

A total of 6.93 × 10^7^ oocysts were recovered. At zero elapsed hours, 100% of oocysts observed were at sporulation stage 1 (completely unsporulated). At 64 h, 93% (95% CI 88–98%) of oocysts observed were at sporulation stage 5 (fully sporulated), and the remaining 7% (2–12%) remained at sporulation stage 1. At both 673 h (35 days) and 835 h, 94% (89–99%) of oocysts were observed at sporulation stage 5, indicating no statistically significant difference in the proportion of oocysts that had completed sporulation between these later time points and at 64 h (*z* = −0.287, *p* = 0.774). Figure 2 shows the percentage of oocysts at each sporulation stage over the course of sporulation. The amount of DNA recovered from time point samples ranged from 37,230 to 416,415 ng total, with A_260/280_ values ranging from 1.69–2.32; Additional File 3: Supplementary Table 3 and Supplementary Fig. 1.

### BLAST searches for qPCR targets

Six putative amplicons of the expected sizes were identified for each of the S1 and S3 primer sets. All were located on chromosome seven (HG994957.1). Four of the predicted amplicons for S1 were identical, one had a single base change (G to C) between the primer anneal sites that was not expected to have major impact on melt temperature, and one showed two base changes in sequential positions (GG to CA) between the primer anneal sites that was predicted to impact melt temperature by ~0.5 °C (via Geneious Prime 2020.2.5, Nucleotide Statistics). All six predicted amplicons for S3 were identical. Of the five areas between repeated S1 or S3 primer hits, four were evenly spaced, with 2.4 kb between a forward primer anneal site for either primer set and the next for the same primer set. The fifth was somewhat larger at 4.4 kb. Ten amplicons were predicted for each of R2 and R3 primer sets. A single target for each primer set occurred on each of chromosomes 10 (HG994970.1) and 11 (HG994971.1), and the remaining eight for each occurred on chromosome 13 (HG994973.1). All predicted R2 amplicons showed 100% sequence identity, as did all predicted R3 amplicons. The targets for R2 and R3 on chromosome 13 were unevenly spaced, with areas between forward primer anneal sites for either primer set ranging from 13.5 to 803.3 kb (Additional File 3: Supplementary Table 4). Three 100% identity BLAST hits were identified for the P1 target: two in the apicoplast genome and a third on chromosome six. No BLAST hits outside of the expected single mitochondrial target for each of primer sets M1 and M2 were identified.

Nuclear Tn-E03-1161 targeting S1 was chosen over S3 for full time point analysis, due to a slightly lower triplicate SD observed in preliminary qPCR runs (data not shown). Primer set R2 was chosen over R3 due to slightly lower SD of same-plate technical triplicates in preliminary analysis (data not shown). Comparison against the mitochondrial genome sequence of *E. tenella* (Guelph strain; GenBank Reference Sequence: PP464288.1) showed two mismatches in the M1 forward primer anneal site; M2 was, therefore, used for complete time point qPCR analysis.

### qPCR assay performance

To assess intrasample variation (replicate well precision), the coefficient of variation for calculated target CNs were determined. Copy number CV did not exceed 12.8% in any sample. To assess plate-to-plate variations in primer performance, C_q_ values generated from SC plasmid wells were interpreted as CNs and the percent accuracy, determined from the calculated target SCN and known SC plasmid copy number. Percent accuracy was 102.0% (95.0% CI 94.0–110.0%) for M2, 107.9% (95.0% CI 99.6–116.2%) for P1, 105.5% (95.0% CI 100.7–110.3%) for R2, and 103.8% (95.0% CI 98.7–108.9%) for S1.

### NGS and read mapping—estimation of relative abundance of organellar genomes

NGS generated 1.09 × 10^7^ to 1.64 × 10^7^ reads per sample, with the percentage of reads that mapped to the *E. tenella* genome having a strong positive correlation with oocyst age [*r*_(6)_ = 1.00, *p* =  < 0.001]; Additional file 3: Supplementary Fig. 1. The depths of coverage used to determine the relative abundances of organellar to nuclear genomes were not sufficient to generate results with acceptable confidence; the data reported are considered as having only value in their support of those data obtained via qPCR. Nuclear genome DOC ranged from 0.2 at 0 h (SD of 0.6) to 8.9 at 835 h (SD of 6.0). Mitochondrial genome DOC ranged from 45.0 at 24 h (SD of 11.6) to 2366.9 at 835 h (SD of 262.1), and DOC of the apicoplast ranged from 8.3 at 0 h (SD of 3.8) to 519.1 at 835 h (SD of 87.8). Relative DOC of the mitochondrial to the nuclear genome was greater early and late in the experiment at 220.2 (95% CI 92.3–348.1) at 0 h and 267.6 (95% CI 224.3–310.9) at 835 h. Relative DOC of the mitochondrial to nuclear genome was lowest at 145.9 (95% CI 85.9–205.9) at 24 h. A similar pattern was observed in the relative DOC of apicoplast versus nuclear genomes, with the highest ratios at 0 and 835 h at a DOC of 37.4 (95% CI −79.4 to 154.3) and DOC of 58.7 (95% CI 25.9–91.5), respectively; the lowest relative DOC of the apicoplast genome occurred at 32 h, at 23.3 (95% CI −10.7 to 57.3). Results of mapping of NGS reads at each of the eight sequenced time points to are summarized in Table 2.

**Table 2:** Mapped next-generation sequencing reads generated from *Eimeria tenella* oocysts during and following the completion of sporulation to the reference genome

Hrs^a^	DOC_Nu_ (SD)	DOC_Mt_ (SD)	DOC_Plas_ (SD)	RDOC_Mt_ (95% CI)	RDOC_Plas_ (95% CI)
0	0.2 (0.6)	48.6 (11.1)	8.3 (3.8)	220.2 (92.3 to 348.1)	37.4 (−79.4 to 154.3)
8	0.3 (0.6)	46.9 (7.8)	10.1 (4.6)	173.3 (105.9 to 240.7)	37.2 (−30.2 to 104.7)
16	0.3 (0.7)	57.4 (10.9)	9.1 (3.9)	195.5 (122.2 to 268.9)	31 (−29.1 to 91.1)
24	0.3 (0.7)	45.0 (11.6)	8.8 (3.9)	145.9 (85.9 to 205.9)	28.4 (−24.2 to 81.0)
32	0.5 (0.8)	72.3 (14.8)	10.6 (5.1)	159.2 (92.5 to 225.9)	23.3 (−10.7 to 57.3)
64	0.8 (1.1)	149.3 (22.3)	21.3 (7.3)	192.5 (135 to 250)	27.5 (−4.6 to 59.7)
381	5.8 (4.1)	1196.2 (140.9)	150.6 (30.5)	205.9 (167.3 to 244.5)	25.9 (10.7 to 41.2)
835	8.9 (6.0)	2366.9 (262.1)	519.2 (87.8)	267.6 (224.3 to 310.9)	58.7 (25.9 to 91.5)

Depth of coverage (DOC) of each of the nuclear (Nu), mitochondrial (Mt), and apicoplast (Plas) genomes was used to calculate relative depth of coverage (RDOC) of each of the organellar genomes to the nuclear genome

^a^Hours elapsed since collection from ceca. 0 h, freshly collected, completely unsporulated; sporulation was morphologically complete at 64 elapsed hours

### NGS read mapping—estimation of 18S rDNA and Tn-E03-1161 copy numbers

Across all time points, SCN of S1 and R2 calculated from NGS read mapping were strongly correlated (*r*_(6)_ = 1.00, *p* =  < 0.001); the average ratio of 18S rDNA copies to Tn-E03-1161 copies was 6.7 (95% CI 6.5–6.9), based on depth of coverage of Tn-E03-1161 relative to the average putative single copy number gene of 9.6 (95% CI 3.6–15.6) and a relative depth of coverage of 18S rDNA of 76.3 (34.7–117.9). Results from BLAST indicated a ratio of 1.7 (from 10 copies of 18S rDNA and six copies of the Tn-E03-1161 sequence). To further investigate the ratio of 18S rDNA to Tn-E03-1161 and to determine the actual SCN of 18S rDNA, all previously mapped reads were pooled and remapped to the 43,741 bp concatenation of the 10 18S rDNA BLAST hits, six Tn-E03-1161 BLAST hits, and three putatively single-copy genes. *HSP90* had a DOC of 14.7 (SD of 4.0), *MIC1* had a DOC of 14.0 (SD of 3.1), and β-tubulin had DOC of 13.7 (SD of 4.5). The DOC for Tn-E03-1161 was 135.9 (SD of 22.7). The DOC for 18S rDNA was 1077.6 (SD of 53.4). Ratios of DOC of Tn-E03-1161 and 18S rDNA to the average DOC of putatively single-copy genes were 9.6 (95% CI 3.6–15.6) and 76.3 (95% CI 34.7–117.9), respectively. The relative DOC of 18S rDNA to Tn-E03-1161 was 7.93 (95% CI 6.6–9.3). Additional information on the estimation of the abundances of the nuclear targets of interest is included in Additional File 2: Supplementary Table 2.

### Quantitative PCR assessment of nuclear target copy numbers

Results from the R2 and S1 primer sets indicated a ratio of 18S rDNA to Tn-E03-1161 of 6.7 (95% CI 6.5–6.9). Quantitative PCR primer sets R3 and S3 were used only in preliminary analyses, as template DNA was limited and R2 and S1 were found to have slightly superior performance. The data generated from these primer sets was investigated; however, for additional support for the ratio of 18S rDNA to Tn-E03-1161 sequences, all combinations of 18S rDNA and Tn-E03-1161-targeting qPCR primer sets indicated ratios that ranged from 6.7 (95% CI 6.5–6.9) to 7.9 (95% CI 7.2–7.6) (Additional File 2: Supplementary Text 4).

### Quantitative PCR assessment of organellar genome copy numbers

**Fig. 3:**
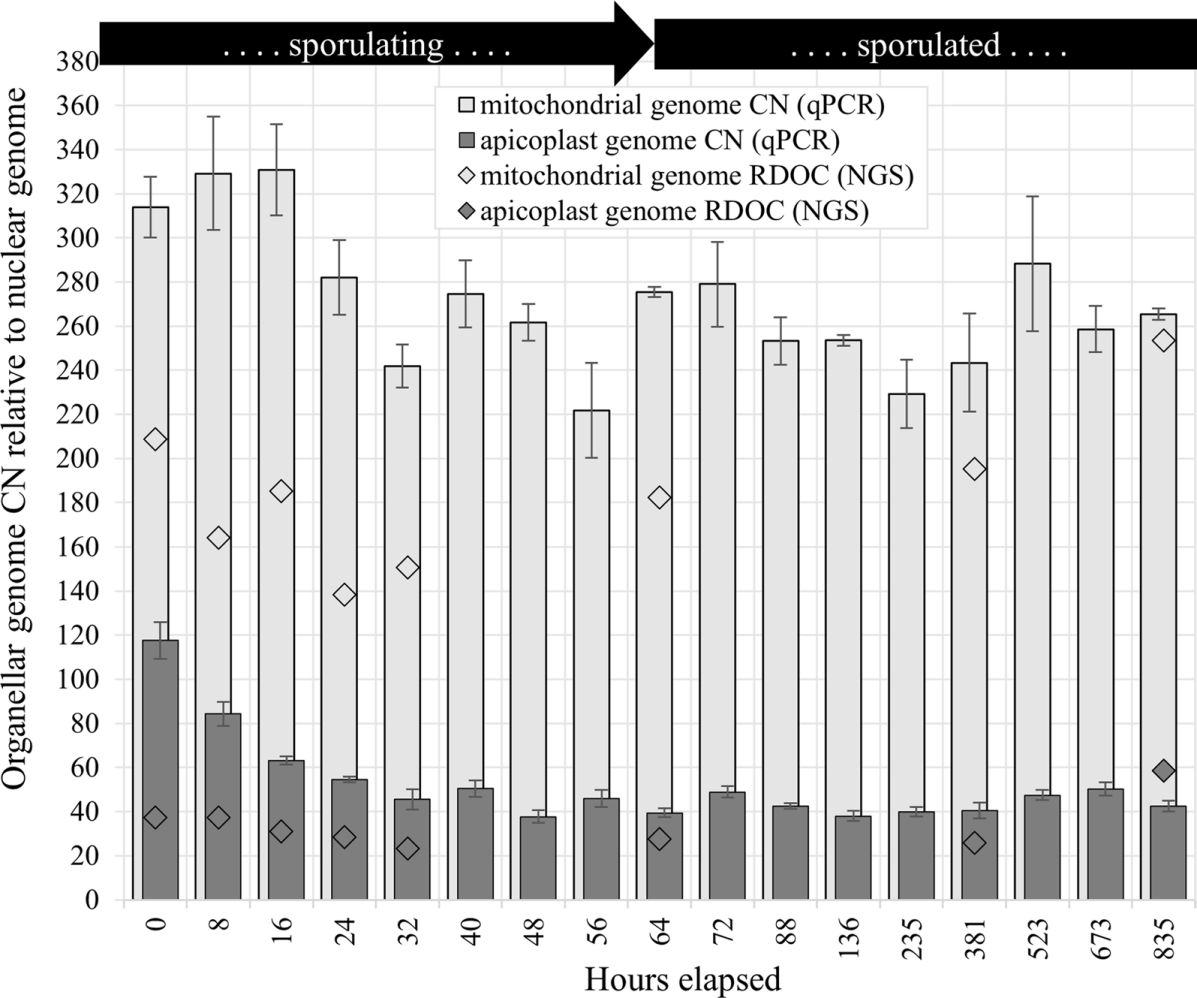
Average copy number (CN) of *Eimeria tenella* mitochondrial (light gray) and apicoplast (dark gray) genomes relative to the nuclear genome during and following the completion of sporulation estimated using qPCR (bars) and relative depth of coverage of whole genome next-generation sequencing (diamonds). Quantitative PCR used primer sets S1 (nuclear target, target copy number of 10), P1 (apicoplast target, target copy number in apicoplast of 2), and M2 (single-copy mitochondrial target). The error bars indicate 95% confidence intervals. No error bars are shown for relative depth of coverage (RDOC) data points, as this method had high relative uncertainty and was not considered of statistical significance in itself; these data shown only as support for qPCR-generated data

**Fig. 4:**
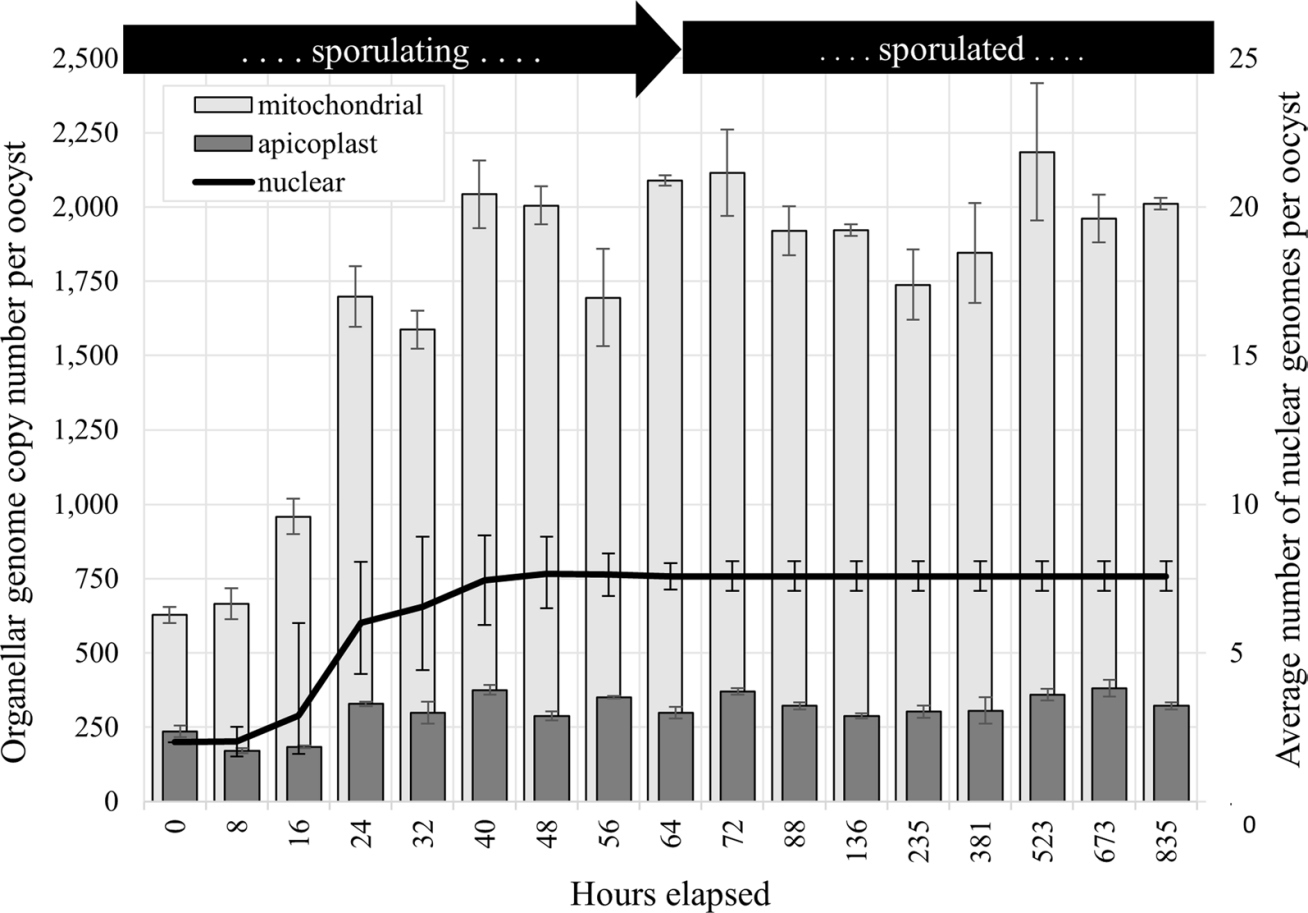
Average copy number (CN) of the mitochondrial genome (light gray, left *y* axis) and the apicoplast genome (dark gray, left *y* axis) per *Eimeria tenella* oocyst during and following sporulation, as determined by qPCR using primer sets S1, P1, and M2. The error bars indicate 95% confidence intervals, calculated from standard deviation of ratios of organellar to nuclear genomes. Estimates of the average number of nuclear genomes per oocyst (black, right *y* axis), were based on proportion of the oocyst population observed with morphology consistent with each sporulation stage; 95% confidence intervals were calculated from standard error of sample proportions

Average organellar genome CNs relative to the nuclear genome throughout the period of the experiment are shown in Fig. 3 and presented in Table 3. Relative SCNs of organellar qPCR target M2 to S1 and of P1 to S1 are shown in Additional file 3: Supplementary Fig. 2. Average nuclear GCN per oocyst was estimated using the proportion of oocysts at each sporulation stage and the expected nuclear GCN associated with each stage (Figs. 1 and 2). Average organellar GCN relative to the nuclear genome at each time point was multiplied by the expected average nuclear GCN per oocyst to determine the each organellar GCN per oocyst; results are shown in Fig. 4.

**Table 3:** Relative abundance of the mitochondrial genome (Mt) and apicoplast genome (Plas) per nuclear genome in sporulating and sporulated oocysts of *Eimeria tenella*, as determined via quantitative polymerase chain reaction using primer sets S1 (target present in ten copies per nuclear genome), M2 (one target copy per mitogenome), and P1 (two target copies per apicoplast genome)

Hours _a_	Mt (95% CI)_b_	Mt shift_c_	Plas (95% CI)_b_	Plas shift_c_
0	313.8 (300.1–327.6)	N/A	117.6 (108.9–126.3)	N/A
8	329.2 (303.4–354.9)	15.3	84.3 (80.5–88.1)	-33.2*
16	330.8 (310.2–351.4)	1.6	63.3 (61.9–64.6)	−21.1*
24	282.0 (265.0–299.0)	−48. 8*	54.5 (53.3–55.8)	−8.7*
32	241.9 (232.1–251.6)	−40.2*	45.6 (39.8–51.4)	−11.8
40	274.6 (259.3–289.8)	32.7	50.4 (48.5–52.3)	7.7
48	261.7 (253.4–270.0)	−12.8	37.7 (35.9–39.4)	−10.8
56	221.8 (200.4–243.3)	−39.9	45.9 (45.4–46.4)	6.3
64	275.5 (273.2–277.7)	53. 7	39.4 (37.2–41.6)	−6.5*
72	279.0 (259.8–298.3)	3.5	48.8 (46.8–50.9)	9.4*
88	253.3 (242.5–264.1)	−25.7	42.5 (41.2–43.9)	−5.7
136	253.6 (251.1–256.0)	0.3	38.0 (36.7–39.3)	−5.2
235	229.3 (213.7–244.9)	−24.3	39.9 (37.5–42.3)	1.9
381	243.4 (221.2–265.6)	14.2	40.4 (37.4–43.4)	2.0
523	288.2 (257.8–318.7)	44.8	47.4 (45.2–49.6)	5.4
673	258.6 (248.1–269.1)	−29.6	50.3 (47.1–53.6)	2.9
835	265.3 (262.8–267.9)	6.7	42.5 (41.1–43.9)	−7.8

Shift in relative abundances of organellar genomes were assessed via one-way analysis of variance (ANOVA), with post hoc paired *t*-tests using *α* = 0.05 to determine shifts in relative abundance from time point to time point. Shifts in relative abundance that were deemed significant are indicated with an asterisk

^a^Hours elapsed since collection of oocysts from ceca

^b^Organellar genome copy number relative to nuclear genome, with 95% confidence interval shown in parentheses

^c^Difference between relative target abundance from previous time point

^*^Statistically significant shifts in genome relative abundance (*p* < 0.05)

### Time point shifts in relative organellar genome copy numbers

A one-way repeated ANOVA was performed to compare the relative abundances of the mitochondrial genome from each successive pair of timepoints. There was a significant effect of elapsed time on mitogenome relative abundance at the *p* < 0.05 level  (*F*(16,2)= 10.4, *p* = 0.029, η^2^ = 0.81). The post hoc paired *t*-test using *α* = 0.05 showed that the mean relative abundance of the mitogenome at 24 elapsed hours (*M* = 282.0, *SD* = 15.0) was significantly different than that at 16 elapsed hours (*M* = 330.8, *SD* = 18.2), *t* (2) = 21.3, *p* = 0.002); and that the mean relative abundance of the mitogenome at 32 elapsed hours (*M* = 241.9, *SD* = 7.6) was significantly different than that at 24 elapsed hours, *t* (2) = 4.4, *p* = 0.048).

Similarly, there was a significant effect of elapsed time on apicoplast relative abundance at the *p* < 0.05 level (*F*(16,2) = 106.8, *p* = 0.002, *η*^2^ = 0.98). The post hoc paired *t*-test using *α* = 0.05 showed five significant shifts in the relative abundance of the apicoplast genome: between 0 h (*M* = 117.6, SD of 7.1) and 8 h (*M* = 84.3, SD of 3.1), *t*(2) = 4.6, *p* = 0.044; 8 h and 16 h (*M* = 63.3, SD of 1.1), *t*(2) = 7.1, *p* = 0.019; between 16 and 24 h (*M* = 54.5, *SD* of 1.0), *t*(2) = 5.8, *p* = 0.029; between 56 (*M* = 45.9, SD of 0.4) and 64 h (*M* = 39.4, SD of 1.8), *t*(2) = 5.5, *p* = 0.032; and between 64 and 72 h (*M* = 48.8, SD of 1.6), *t*(2) = 17.3, *p* = 0.003.

## Discussion

Sporulation involves synthesis of a complete complement of organelles for each of the eight daughter sporozoites [4, 43]. Each zoite possesses a single mitochondrion and a single apicoplast [8, 44–46], each with its own genome, which is present in multiple copies [47–53]. Apicoplast and mitochondrial genomes have been sequenced in dozens of apicomplexan species, including *E.* *tenella* [13, 54]. Reports of organellar GCN have varied across apicomplexan species and stages. Apicoplast GCNs range from five to 25 in *T. gondii* [22, 55], while reports of mitochondrial GCN range from 15 (in *Plasmodium gallinaceum* [56]); to 450 (in *T. gondii* [22]). In sporulated *Eimeria falciformis* oocysts, NGS-based estimates for the apicoplast and mitochondrial GCNs are 18 and 180, respectively [57]*.* Variation in reported organellar GCN may be due to differences in metabolic requirements between different species and different lifecycle stages within a single species. It is also important to note, however, that all of these reported GNC were determined incidentally in studies focused on other objectives using semiquantitative protocols; this may also contribute to variations in reported GCN.

Mitochondrial cytochrome *c* oxidase subunit I (*COI*) and subunit III (*COIII*), both present in single copies in the mitochondrial genome of *Eimeria* spp., have become common loci used in species delimitation and phylogenetic inference [58, 59]. Recently, mitochondrial *COIII* was proposed as the target of a digital droplet polymerase chain reaction (ddPCR)-based assay for the determination of relative abundances of *Eimeria* spp. infecting chickens in mixed oocyst samples [60]. The real-world application of such an assay is in the monitoring of coccidiosis management programs and the tailoring of vaccination protocols—two important requirements in the improved sustainability and capacity of industrial food animal production. For such qualitative assays to be truly applicable, more accurate knowledge of the mitochondrial GCN in oocysts of *Eimeria* spp. is required.

The A/T-rich apicoplast genome contains a high density of genes encoding proteins, rRNA, and tRNA [61]; these characteristics render this organellar genome a less appropriate target for quantitative assays [62]. Despite this, the presented data may serve to contribute to improving understandings of the basic biology of the eimerian apicoplast. Given the indispensable nature of the organelle [63], this area merits ongoing investigation.

Previous reports suggest that sporulation of *E.* *tenella* oocysts requires approximately 48 h under optimal conditions (high humidity, 24–28 °C) [64]. We observed morphological progress of sporulation was completed between 56 and 64 h, with 93% of oocysts completing sporulation. This slightly longer time to complete sporulation was likely due to the lower than optimal sporulation temperature (21 °C), while the percentage of oocysts successfully completing sporulation was in line with a previous report [30]. On average, more DNA was recovered from oocyst subsamples taken at earlier time points; however, the proportion of DNA attributed to *E. tenella* (reflected by the percent of NGS reads mapped) showed a strong positive correlation with elapsed time (*r*_(6)_ = 1.00, *p* < 0.001). Decay of bacterial, host, and other environmental DNA in the stored oocyst samples; change in efficiencies of oocyst disruption and DNA recovery as a function of time; and, possibly, decay of the ~7% of nonviable oocysts (those that did not sporulate) may have contributed to the relationship observed between the amount and quality of DNA recovered and the passage of time.

Data generated by qPCR at all timepoints indicated a ratio of ~6.7 copies of 18S rDNA per copy of the Tn-E03-1161 sequence, in stark disagreement with the ratio of ~1.7 determined via BLAST query. NGS read mapping suggested an 18S rDNA SCN of 76.3 (95% CI 34.7–117.9), a number that is in line with estimates of copy number of this sequence in other apicomplexan parasites that range from 110 in *T.* *gondii* [65] to single digit CNs, such as five, in *Cryptosporidium parvum* [66] and eight in *P. falciparum* [67]. NGS read mapping supported use of a S1 SCN of 10 for determination of relative abundances of other targets of interest. Inaccuracies in the estimated Tn-E03-1161 SCN would impact reported relative SCNs of other qPCR targets and of organellar GCNs, proportionately. Further work should include qPCR primer sets targeting both Tn-E03-1161 and several putatively single-copy genes to provide greater frame of reference for absolute target SCNs.

All shifts in relative abundance of the apicoplast genome up until 24 h, and those between 56 and 72 h were found to be statistically significant via one-way repeated measures ANOVA with a post hoc paired *t*-test using *α* = 0.05 (Table 3). No morphological indication of sporulation activities beyond 64 h could be observed microscopically; the shift in abundance of the apicoplast genome, relative to a single copy of the nuclear genome, from 39.4 copies (95% CI 37.2–41.6) at 64 h to 48.8 copies (46.8–50.9) at 72 h (*p* = 0.003) suggests that ongoing apicoplast DNA synthesis occurs for a short time after the formation of daughter sporozoites. This is in contrast with previous reports based on *T. gondii*, which indicate apicoplast genome replication is completed prior to the beginning of replication of the nuclear genome [49, 68]. The oocyst is shed with 25% of the nuclear DNA that will be present in the eight sporozoites resulting from its sporulation; the data reported here suggest that at the time of shedding, the oocyst possesses ~60% of the apicoplast genetic material that will be present at 72 elapsed hours (from ~235 copies to ~390 copies).

Relative abundance of the mitochondrial genome showed a high degree of variation. Only two shifts in the relative SCN of M2 were found to be statistically significant via one-way ANOVA with a post hoc paired *t*-test using *α* = 0.05 (Table 3): from 330.8 (95% CI 310.2–351.4) at 16 elapsed hours to 282.0 (265.0–299.0) at 24 h (*p* = 0.002) and from 282.0 at 24 h to 241.9 (232.1–251.6) at 32 h (*p* = 0.048). Data suggest that at the time of shedding, oocysts possessed ~28% of the mitochondrial genetic material that was present at the morphological completion of sporulation (from ~628 copies to ~2204 copies). The data reflecting the mitochondrial genome relative abundance may be obscured by the semi-asynchronous sporulation of the oocyst stock. If reports of mitochondrial genome replication beginning alongside and being completed before that of the nuclear genome of *P. falciparum* [19] hold true in *E.* *tenella*, then little variation in relative abundance of the mitochondrial genome to that of the nuclear genome would be expected. The variation in the data shown here may simply reflect a relatively wide range of true biological variation in the abundance of this organellar genome.

Future work should use probe-based qPCR, which would allow the measurement of abundance of two targets per genome for all three genomes in a single well. This would improve assay accuracy (by minimizing opportunity for experimenter error and allowing for purely relative quantification) and would significantly reduce the amount of DNA template required [69–71], possibly to the point at which analyzing DNA from a single oocyst at a time may be possible. A single-oocyst qPCR would remove the challenge presented by nonsynchronously sporulating oocysts, but accomplishing reliable DNA extraction from all compartments in the sporulating oocyst is technically challenging because of the nature of these oocysts. The inclusion of additional time points between 0 and 72 elapsed hours could help to improve the resolution of the picture painted by the data presented here. Finally, further work toward describing organellar genome abundances relative to the nuclear genome in endogenous stages of the lifecycle would be a valuable expansion of scope and would remove the challenge of extracting DNA from rugged oocyst structures.

## Conclusions

Sporulation, the phase of the endogenous portion of the lifecycle of *Eimeria* spp. during which parasites become infectious to the host, is relatively poorly understood in part due to the technical difficulty of obtaining sufficient high quality parasite material for proteomic, genomic or similar investigations. The work described here contributes to basic understandings of the biology of these parasites and raises several questions pertaining to the cell cycle of *E. tenella*. Results suggest that some degree of apicoplast DNA synthesis occurs after 64 h, which marks the morphological completion of sporulation; however, no significant shifts in relative abundance of either organellar genome occurred after 72 h. Further, our data suggest that organellar genome replication is not characterized by simple doubling events, with measured abundances at the completion of sporulation not aligning with what would result from single or successive doublings of the organellar genomic material present in unsporulated oocysts. Finally, by determining the actual SCN of nuclear 18S rDNA and Tn-E03-1161 sequences, the usefulness of these targets in parasite quantification assays or as normalizers in molecular work is expanded. That the existing full reference genome [37] dramatically underestimates the SCN of this gene indicates a need for ongoing refinement of these chromosomal sequences.

## Supplementary Information

**Additional file 1: Supplementary Text 1.** Quantitative polymerase chain reaction (qPCR) primer validation. **Supplementary Text 2.** Generation of plasmid for use in primer validation and as qPCR control. **Supplementary Table 1.** Standard-curve determined characteristics of qPCR primer performances. **Supplementary Formula S1.** Copy number calculation from quantification cycle (C_q_).

**Additional file 2: Supplementary Text 3.** Putatively single-copy nuclear genes used in estimating copy number of 18s rDNA and Tn-E03-1161 sequences in *Eimeria tenella*. **Supplementary Formula 2.** Average depth of coverage (DOC) formula. **Supplementary Table 2.** Putatively single-copy nuclear genes and relative depth of coverage calculations for estimation of nuclear 18S rDNA and Tn-E03-1161 copy numbers in *Eimeria tenella*. **Supplementary Text 4.** Further investigation of the ratio of 18S rDNA to Tn E03 1161.

**Additional file 3: Supplementary Table 3.** DNA recovered from *Eimeria tenella* oocyst timepoint samples. **Supplementary Figure 1.** DNA recovered from oocyst timepoint samples and % NGS reads mapped per sample. **Supplementary Table 4.** Nuclear-targeting qPCR primer BLAST results. **Supplementary Figure 2.** Ratios of mitochondrial and apicoplast to nuclear qPCR targets in sporulating and sporulated oocysts of *Eimeria tenella*.

## Data Availability

The NGS datasets supporting the conclusions of this article are available in the Mendeley Data repository under 10.17632/xf6rggrsn6.1 (NGS at 0 elapsed hours of sporulation and 8 elapsed hours); 10.17632/547wwd5jfr.1 (NGS at 16 elapsed hours of sporulation and 24 h elapsed); 10.17632/962ypgjnmj.1 (NGS at 32 elapsed hours of sporulation and 64 h elapsed); 10.17632/frxnm8vyys.1 (NGS at 381 elapsed hours of sporulation and 835 h elapsed).

## Abbreviations

CI: Confidence interval
CN: Copy number
DOC: Depth of coverage
NGS: Next-generation sequencing
qPCR: Quantitative polymerase chain reaction
SD: Standard deviation
