# Supplementary material for: Organellar genome dynamics of exogenous stages of Eimeria tenella
Source: Parasit Vectors. 2024 Oct 13;17:428. doi: 10.1186/s13071-024-06498-w (PMC11476305; doi:10.1186/s13071-024-06498-w)
Supplement: Supplementary file 1 — Additional file 1: Supplementary Text 1. Quantitative polymerase chain reaction (qPCR) primer validation. Supplementary Text 2. Generation of plasmid for use in primer validation and as qPCR control. Supplementary Table 1. Standard-curve determined characteristics of qPCR primer performances. Supplementary Formula S1. Copy number calculation from quantification cycle (Cq). [file 13071_2024_6498_MOESM1_ESM.docx]

**Supplementary Text 1: Quantitative polymerase chain reaction primer validation**

Quantitative PCR primers were initially assessed by standard PCR, which included 1.25 U of Platinum Taq DNA polymerase (Thermo Fisher Scientific), 1× PCR buffer (Thermo Fisher Scientific, Waltham, MA), 0.2 mM each deoxynucleotide, 3 mM MgCl_2_, 0.2 μM both forward and reverse primer, and 50 ng DNA from the experimental timepoint sample taken at 381 total elapsed hours. Total PCR volume was 25 μL. Amplification products were separated electrophoretically through a 1.5% submarine agarose gel prepared with 1× Tris-acetate EDTA (TAE) buffer and containing 40 ng/ml ethidium bromide. Reaction product bands were visualized using UV transillumination and approximate amplicon sizes were determined by comparison to a 100 to 1,000 bp DNA marker (Bio Basic Inc., Mississauga, Ontario, Canada) to confirm presence of a single band of the expected size.

To establish standard curves for each qPCR primer set, quadruplicate qPCRs were run using a series of 10-fold dilutions of cloned control plasmid as template (ranging from 2.76 × 10^8^ to 276 copies; see below). Briefly, a total reaction volume of 10 µL contained 1× PowerUP^TM^ SYBR Master Mix (Thermo Fisher Scientific), 600 nM forward primer and 600 nM reverse primer, nuclease-free water, and 1.5 μL template (approximately 15 ng). Reactions were carried out on a QuantStudio™ 7 Pro Real-Time PCR System (Applied BiosystemsTM, Waltham, MA) with MicroAmp™ EnduraPlate™ Optical 384-Well Clear Reaction Plates (Applied BiosystemsTM) via the following cycling conditions: uracil-DNA glycosylase deactivation at 50^o^C for 2 minutes, Dual-lock DNA polymerase activation at 95^o^C for 2 minutes; followed by 40 cycles of: template denature at 95^o^C for 15 seconds and a combined anneal/extend step at 60^o^C for 1 minute. The melt program consisted of 95^o^C for 15 seconds (ramp rate: 1.6^o^ /second), 60^o^C for 1 minute (ramp rate: 1.6^o^C/second) and ending with 95^o^C for 15 seconds (ramp rate: 0.15^o^C/second). Thermocycler cover temperature was set to 105^o^C. Each primer set was run on a series of two technical replicates on separate plates. Quantification cycle (C_q_) values were originally determined using the Design & Analysis (Applied Biosystems) software’s auto threshold setting, using ROX as passive reference.

Resulting C_q_ values were graphed against the logarithmic value of the qPCR target copy number to determine the regression formula that related C_q_ value to copy number, and the slope and y-intercept for each primer set.

**Supplementary Text 2: Generation of plasmid for use in primer validation and as qPCR control**

A control sequence containing a single copy of each of the qPCR targets used in the presented work, in addition to several others used in unrelated work, was manually designed using Geneious Prime (2020.2.5) and synthesized as a gBlock^TM^ Gene Fragment by Integrated DNA Technologies (IDT; Coralville, Iowa). The control sequence was inserted into a pJET1.2/blunt vector using a CloneJET PCR Cloning Kit (Thermo Scientific™). OneShot^TM^ TOP10 chemically competent *E. coli* cells (Invitrogen, Burlington, ON) were transformed with the construct-containing plasmid. Plasmids were recovered from overnight growth in LB using GeneJET Plasmid Midiprep Kit (Thermo Scientific™). All protocols were as per the manufacturer’s instructions. Plasmid sequence identity was confirmed via Sanger sequencing. The plasmid was linearized via restriction endonuclease digest and a 10-fold dilution series was generated for use in standard curve construction and characterization of qPCR performance. Each dilution contained a background concentration of wrong-template control plasmid (pJET1.2 vector that contained *Eimeria adenoides* partial mitochondrial COI insert, generated with primers COI_UNI_199F [ATGATYTTCTTTGTAGTTATGCC] and mtRNA20_UNI_R [GTATGGATTTCACGGTCAA]; amplicon = 1,272 bp) at a concentration of 10 ng/μL.

**Control plasmid insert sequence:** The control plasmid consisted of the designed gBlock^TM^ insert within the pJET1.2 vector. The insert sequence was as follows:

1 atacctctgg tctagacgac tagaatctgc gaggcccagt tcaagaaggt caaagaggac

61 cgcgagggcg caatggctgc tgctgggcgc ctgcatgcgg tgctgaagcg gctgcaggag

121 aaggctgctg ctgggctgct gctgtggcag cgcgagcacg gcgcagcagc agctgcagca

181 gcagatggac aagaagagga aaatgcttca attgaaatcc gagtcaacgc tgggagatcg

241 tcaggtgatt cgaaaagaag catatgtcag ccaagccgtc ataaaggaac ttagaagaat

301 tattgaagag gcagaaatcc tgcgcgaaga cgacaacaac tcacatcgtc gagaagatcg

361 acagccacat gtaccacaag ctgtacgtac gctgcaaagg caaccagttc aaaaacaaac

421 gcgtgctgat cgaagccatt cacaatgaaa agaacttgaa ggtgaaggag aaggcgctgc

481 aggagcaggt ggactctgca tacctggtgt gccgcatggg catcaacggc ttcggccgca

541 ttgggcggtt ggtcttccgt gccgcaatgg ccaacccaaa tgtggaggtc gttgcgctga

601 acgatccctt catggatgtc cagtatatgg cttaccagct gaagttcgac tccgtccacg

661 gcagatatcc tggagaagtg acagtgaagg acggcaacct ggttgttgta gttaagcttt

721 tataaatgcg tcttctctgg cgacgagctc tgctcggatt cataccctca tctggtgccg

781 ttcgatgatg acgcgttcaa ggacgttgcc tttgaggtga agggaaccac agtctggaaa

841 attcaaagtg cgttgctagt accaacccta ttttcattct tcgccaaaag cactctgatt

901 gacaaatacg atttatctaa tttacacgaa attgcttctg ggggcgcacc tctttcgaaa

961 gaagtcgggg aagcggttgc aaaatacggt atcgtaaggc tatgaagaga tacgccctgg

1021 ttcctggaac aattgctttt acagatgcac atatcgaggt gaacatcacg tacgcggaat

1081 acttcgaaat gtccgttcgg ttggcagaag ctatgaaacg atatgttgtg cttataagct

1141 cctctgggtt cgtgttgttg cattagagaa tcaaaatttc tacaacctag cattcacact

1201 gcatggtgct attatgattt tctttgtagt tatgccaggt ctttttggtg gatatggtaa

1261 ggataatgca aagtatccct gactggattc tgcaaaaact aattttttga gaacagcttc

1321 caagcataca tttgttgatg ttatagtaga attcatattt tattgcctgt caagttcctt

1381 taatgtagtt atctcacagc ttctcttggt ccagataagc gatgatcaga acaagctgac

1441 gacacccgta ctaaaactga cacaagtaag caagtataaa tatactaagg agtacgaaat

1501 aactttttct aaggaactcg gcaaaataat cctgtaatcc accgcttcac agtaggcaac

1561 agtgcgaaac tcaccaggtc cagacatggg aaggattgac agattgatag ctctttcttg

1621 attctatggg tggtggtgca tggccgttct tagttggtgg agtgatctgt ctggttaata

1681 gtctactctg gcatgccctc ggcttatttc cggtagcctt ccgcgcttaa ttgcgtgtgt

1741 tggtgttctg gaacttttac tttgagaaaa atagagtgtt tcaagcaggc ttgtcgccct

1801 gaatactgca gcatggaata ataagatagg agctgtaatc gagcaagaca cgcgttgttg

1861 gctatcgtag gcccttcaga ggcaggaagc gcttctctgt cataaacgtt catcttccgt

1921 ccactcttga atagcatgga agactgagca tggccggcta tgaagcacct gtgtactgcg

1981 tgaggtacca agaccatacg tgaatgaacg ctattgccga ctacagcggg aggtgtctca

2041 acagactccg gataggtcga gg

**Supplementary Table 1**

Characteristics of qPCR primer performance, as determined via standard curve establishment.

| Target | Amplicon length (bp) | % Efficiency | Slope | *y*-Intercept |
| --- | --- | --- | --- | --- |
| R2 | 100 | 100.3 | -3.32 | 35.78 |
| R3 | 124 | 101.6 | -3.29 | 35.403 |
| S1 | 76 | 103.1 | -3.25 | 35.97.0 |
| S3 | 69 | 101.3 | -3.29 | 36.62 |
| M1 | 105 | 99.8 | -3.33 | 36.90 |
| M2 | 143 | 100.2 | -3.32 | 35.30 |
| P1 | 95 | 102.4 | -3.27 | 35.35 |

**Supplementary Formula 1**

*qPCR target sequence copy number = 10^[Cq – y intercept]/slope^*
