# Supplementary material for: Organellar genome dynamics of exogenous stages of Eimeria tenella
Source: Parasit Vectors. 2024 Oct 13;17:428. doi: 10.1186/s13071-024-06498-w (PMC11476305; doi:10.1186/s13071-024-06498-w)
Supplement: Supplementary file 2 — Additional file 2: Supplementary Text 3. Putatively single-copy nuclear genes used in estimating copy number of 18s rDNA and Tn-E03-1161 sequences in Eimeria tenella. Supplementary Formula 2. Average depth of coverage (DOC) formula. Supplementary Table 2. Putatively single-copy nuclear genes and relative depth of coverage calculations for estimation of nuclear 18S rDNA and Tn-E03-1161 copy numbers in Eimeria tenella. Supplementary Text 4. Further investigation of the ratio of 18S rDNA to Tn E03 1161. [file 13071_2024_6498_MOESM2_ESM.docx]

**Supplementary Text 3:** **Putatively single copy nuclear genes used in estimating copy number of 18s rDNA and Tn-E03-1161 sequences in *Eimeria tenella***

Putatively single copy genes included: *HSP90* (heat shock protein 90), described as single copy in both *Eimeria acervulina* [40] and *Eimeria tenella* [40, 39]. Messenger RNA sequence AF042329.1 was used to identity the CDS on chromosome 7 (HG994967.1) of the *E. tenella* reference genome [37]. Βeta‑tubulin, described as single copy in *E. tenella* [41]. Messenger RNA sequence U19609.1 was used to identify the CDS on chromosome 4 (HG994964.1) of the *E. tenella* reference genome. *MIC1* (microneme protein 1), described as single copy in *Eimeria maxima* [42]. Messenger RNA sequence KF791866.1 was used to identify the CDS on chromosome 13 (HG994973.1) of the *E. tenella* reference genome.

**Formula 2: NGS read mapping depth of coverage**

| *DOC =* | *# mapped reads × average read length* |  |
| --- | --- | --- |
|  | *length of reference sequence in base pairs* |  |

**Supplementary Table 2 - Putatively single copy nuclear genes and relative depth of coverage calculations for estimation of nuclear 18s rDNA and Tn-E03-1161 copy numbers in *Eimeria tenella***

| Reference sequence (length _a_) | Raw read count | Depth of coverage _b_ | | Standard deviation | | Copy number based on SC references |
| --- | --- | --- | --- | --- | --- | --- |
| SC: B-tubulin (2,088_)_ | 109 | 13.7 | SC avg = 14.1 | 4.5 | SC pooled = 3.9 | 1 |
| SC: *HSP90* (3,026) | 169 | 14.7 |  | 4.0 |  |  |
| SC: *MIC1* (2,548) | 136 | 14.0 |  | 3.1 |  |  |
| Tn-E03-1161 (1,161) | 124.7; 47.8; 62.6; 136.1; 131.1; 98.6  (601 total) | 135.9 | | 22.7 _c_ | | 9.6  (95% CI = 3.6 - 15.6) |
| 18S rDNA (1,756) | 526.8; 759.0; 803.9; 734.3; 637.9; 818.4; 664.2; 709.7; 932.3; 619.5 (7206 total) | 1,077.6 | | 53.4 _c_ | | 76.3  (95% CI = 34.7 - 117.9) |

_a_ Reference sequence lengths reported in bp; do not include the additional 300 base up- and downstream

_b_ Depth of coverage calculated from read count, average read length of 262.2, and length of reference sequence

SC = single copy reference sequence(s)

_c_ Standard deviation of depth of coverage of 18S rDNA and Tn-E03-1161 calculated from re-map to consensus with 15% mismatches permitted

**Supplementary Text 4: Further investigation of the ratio of 18S rDNA to Tn‑E03‑1161**

Basic logical alignment search tool (BLAST) queries of the published *E. tenella* reference [37] (GenBank #: HG994961.1–HG994977.1) indicated a ratio of 1.7 for the ratio of 18S rDNA copies to the Tn‑E03‑1161 sequence. Mapping of pooled Next Gen sequencing (NGS) reads to target sequences observed in the reference genome suggested a ratio of 18S rDNA copies to the Tn‑E03‑1161 sequence of 7.9 (95% CI = 6.5–9.3). Ratios of copies of *Eimeria tenella* 18S rDNA to copies of the Tn‑E03‑1161 sequence were calculated from sequence copy number values calculated from 18S rDNA targeting primer sets R2 and R3, and from Tn‑E03‑1161 targeting primer sets S1 and S3. Results from the R2 and S1 primer sets indicated a ratio of 6.7 (6.5–6.9); results from R3 and S1 indicated a ratio of 7.1 (6.9–7.3); results from R2 and S3 indicated a ratio of 7.9 (7.5–8.3); results from R3 and S3 indicated a ratio of 7.4 (7.2–7.6).
