# Supplementary material for: Organellar genome dynamics of exogenous stages of Eimeria tenella
Source: Parasit Vectors. 2024 Oct 13;17:428. doi: 10.1186/s13071-024-06498-w (PMC11476305; doi:10.1186/s13071-024-06498-w)
Supplement: Supplementary file 3 — Additional file 3: Supplementary Table 3. DNA recovered from Eimeria tenella oocyst timepoint samples. Supplementary Figure 1. DNA recovered from oocyst timepoint samples and % NGS reads mapped per sample. Supplementary Table 4. Nuclear-targeting qPCR primer BLAST results. Supplementary Figure 2. Ratios of mitochondrial and apicoplast to nuclear qPCR targets in sporulating and sporulated oocysts of Eimeria tenella. [file 13071_2024_6498_MOESM3_ESM.docx]

**Supplementary Table 3: DNA recovered from *Eimeria tenella* oocyst timepoint samples**

DNA recovered from oocyst subsamples during sporulation and following its completion.

| Hours elapsed | Days elapsed | Ng/μL | Total ng | A_260/280_ |
| --- | --- | --- | --- | --- |
| 0 _a_ | 0.0 | 344.2 | 292,570 | 2.03 |
| 8 | 0.3 | 489.9 | 416,415 | 1.92 |
| 16 | 0.7 | 485.6 | 412,760 | 2.00 |
| 24 | 1.0 | 245.0 | 208,250 | 2.00 |
| 32 | 1.3 | 378.8 | 321,980 | 1.99 |
| 40 | 1.7 | 294.2 | 250,070 | 1.98 |
| 48 | 2.0 | 355.4 | 302,090 | 1.97 |
| 56 | 2.3 | 402.8 | 342,380 | 2.08 |
| 64 _b_ | 2.7 | 304.0 | 258,400 | 1.91 |
| 72 | 3.0 | 348.9 | 296,565 | 2.11 |
| 88 | 3.7 | 204.2 | 173,570 | 1.95 |
| 136 | 5.7 | 334.9 | 284,665 | 1.99 |
| 235 | 9.8 | 142.4 | 121,040 | 1.95 |
| 381 | 15.9 | 117.5 | 99,875 | 1.87 |
| 523 | 21.8 | 161.9 | 137,615 | 2.32 |
| 673 | 28.0 | 209.9 | 178,415 | 2.24 |
| 835 | 34.8 | 43.8 | 37,230 | 1.69 |

_a_ At 0 elapsed hours completely unsporulated oocysts had just been recovered from cecae

_b_ At 64 elapsed hours sporulation was complete

**Supplementary Figure 1:** **DNA recovered from oocyst timepoint samples and % NGS reads mapped per sample**

Total DNA was recovered from samples of 7 × 10^5^ *Eimeria tenella* oocysts. NGS was only conducted at time elapsed = 0, 8, 16, 24, 32, 64, 381, and 835 hours.


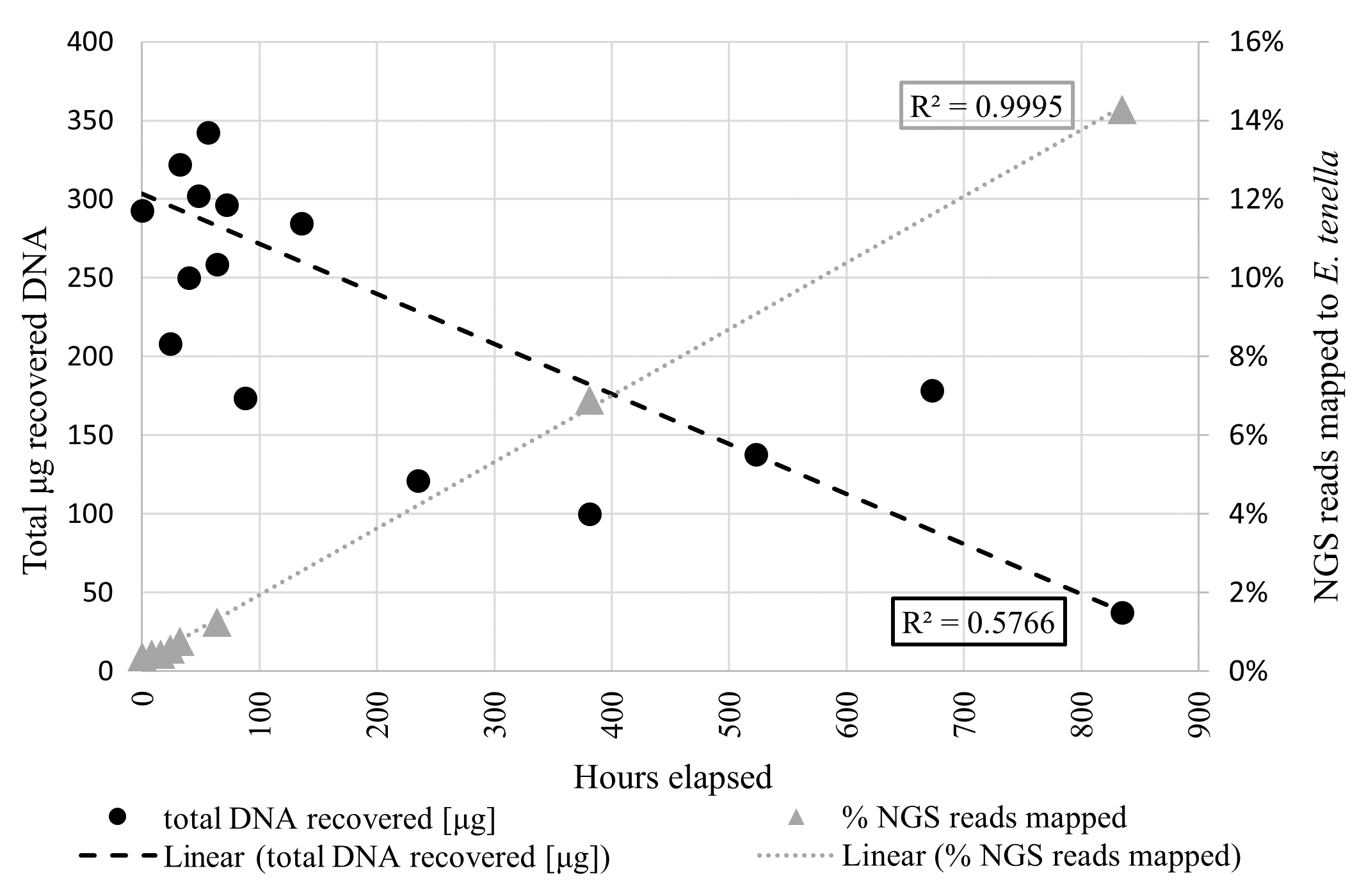


**Supplementary Table 4: Nuclear-targeting qPCR primer BLAST results**

Occurrences of qPCR targets in the nuclear genome of *Eimeria tenella*. Chr = chromosome; F/R bind = forward/ reverse primer bind site - start and end positions correspond to the original numbering of the full chromosome sequence; Amp. Size = amplicon size (bp). Unless noted otherwise, all amplicons predicted for a single primer set showed 100% sequence identity.

| Target | Accession | Chr. | F bind | R bind | Notes |
| --- | --- | --- | --- | --- | --- |
| S1 | HG994957.1 | 7 | 42,363 | 42,288 | None. |
|  | HG994957.1 | 7 | 44,767 | 44,692 | F primer anneals 2,404 bp downstream of previous F primer anneal site for same primer set. In-amplicon G 🡪 C, not predicted to significantly impact product T_m_. |
|  | HG994957.1 | 7 | 47,171 | 47,096 | F primer anneals 2,404 bp downstream of previous F primer anneal site for same primer set. |
|  | HG994957 | 7 | 49,575 | 49,500 | F primer anneals 2,404 bp downstream of previous F primer anneal site for same primer set. |
|  | HG994957 | 7 | 51,979 | 51,904 | F primer anneals 2,404 bp downstream of previous F primer anneal site for same primer set. |
|  | HG994957 | 7 | 54,391 | 54,316 | F primer anneals 2,412 bp downstream of previous F primer anneal site for same primer set. In-amplicon GG 🡪 CA, predicted to impact product T_m_ by ~0.5^o^ C |
| S3 | HG994957 | 7 | 43,162 | 43,094 | None. |
|  | HG994957 | 7 | 45,566 | 45,498 | F primer anneals 2,404 bp downstream of previous F primer anneal site for same primer set. |
|  | HG994957 | 7 | 47,970 | 47,902 | F primer anneals 2,404 bp downstream of previous F primer anneal site for same primer set. |
|  | HG994957 | 7 | 50,374 | 50,306 | F primer anneals 2,404 bp downstream of previous F primer anneal site for same primer set. |
|  | HG994957 | 7 | 52,778 | 52,710 | F primer anneals 2,404 bp downstream of previous F primer anneal site for same primer set. |
|  | HG994957 | 7 | 55,190 | 55,122 | F primer anneals 2,412 bp downstream of previous F primer anneal site for same primer set. |
| R2 | HG994970 | 10 | 241,620 | 241,743 | None. |
|  | HG994971 | 11 | 3,390,480 | 3,390,579 | None. |
|  | HG994973 | 13 | 2,348,126 | 2,348,225 | None. |
|  | HG994973 | 13 | 2,943,580 | 2,943,679 | F primer anneals 595,454 bp downstream of previous F primer anneal site for same primer set. |
|  | HG994973 | 13 | 2,957,080 | 2,957,159 | F primer anneals 13,500 bp downstream of previous F primer anneal site for same primer set. |
|  | HG994973 | 13 | 3,760,418 | 3,760,517 | F primer anneals 808,338 bp downstream of previous F primer anneal site for same primer set. |
|  | HG994973 | 13 | 3,778,790 | 3,778,889 | F primer anneals 18,372 bp downstream of previous F primer anneal site for same primer set. |
|  | HG994973 | 13 | 3,799,567 | 3,799,666 | F primer anneals 20,777 bp downstream of previous F primer anneal site for same primer set. |
|  | HG994973 | 13 | 3,832,558 | 3,832,657 | F primer anneals 32,991 bp downstream of previous F primer anneal site for same primer set. |
| R3 | HG994970 | 10 | 242,136 | 242,235 | None. |
|  | HG994971 | 11 | 3,389,964 | 3,390,087 | None. |
|  | HG994973 | 13 | 2,347,610 | 2,347,733 | None. |
|  | HG994973 | 13 | 2,943,064 | 2,943,187 | F primer anneals 595,454 bp downstream of previous F primer anneal site for same primer set. |
|  | HG994973 | 13 | 2,957,695 | 2,957,572 | F primer anneals 14,631 bp downstream of previous F primer anneal site for same primer set. |
|  | HG994973 | 13 | 3,761,033 | 3,760,910 | F primer anneals 808,338 bp downstream of previous F primer anneal site for same primer set. |
|  | HG994973 | 13 | 3,779,405 | 3,779,282 | F primer anneals 18,372 bp downstream of previous F primer anneal site for same primer set. |
|  | HG994973 | 13 | 3,800,182 | 3,800,059 | F primer anneals 20,777 bp downstream of previous F primer anneal site for same primer set. |
|  | HG994973 | 13 | 3,833,173 | 3,833,050 | F primer anneals 32,991 bp downstream of previous F primer anneal site for same primer set. |
|  | HG994973 | 13 | 3,858,768 | 3,858,645 | F primer anneals 25,595 bp downstream of previous F primer anneal site for same primer set. |

**Supplementary Figure 2:** **Ratios of mitochondrial and apicoplast to nuclear qPCR targets in sporulating and sporulated oocysts of *Eimeria tenella***

Ratios of apicoplast (dark grey) and mitochondrial (light grey) qPCR targets P1 and M2, relative to nuclear target S1. Statistically significant shifts in relative abundance are shown with a dashed line. Error bars indicate 95% confidence intervals based on standard deviation of organellar to nuclear target ratios calculated from replicate qPCR wells. Note that reported P1 target abundance has been adjusted to reflect only apicoplast targets (i.e. not the single nuclear copy).

**
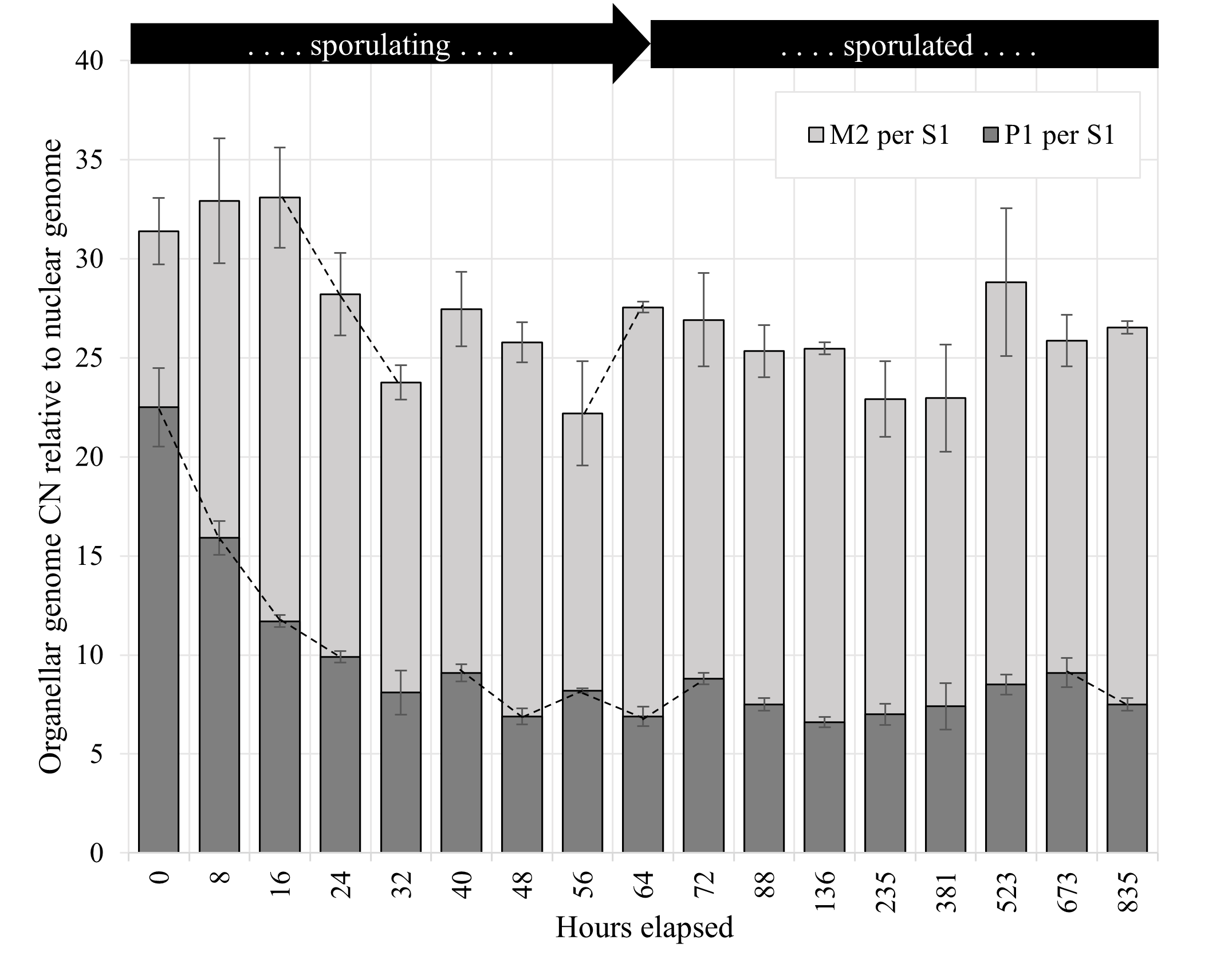
**
